# Uric Acid Levels Are Associated with Bone Mineral Density in Mexican Populations: A Longitudinal Study

**DOI:** 10.3390/nu14204245

**Published:** 2022-10-12

**Authors:** Karina Robles-Rivera, Anna D. Argoty-Pantoja, Alberto Hidalgo-Bravo, Amado D. Quezada-Sánchez, Guadalupe León-Reyes, Yvonne N. Flores, Jorge Salmerón, Rafael Velázquez-Cruz, Berenice Rivera-Paredez

**Affiliations:** 1 Research Center in Policies, Population and Health, School of Medicine, National Autonomous University of Mexico (UNAM), Mexico City 04510, Mexico; 2 Department of Genetics, National Institute of Rehabilitation (INR), Mexico City 014389, Mexico; 3 Center for Evaluation and Surveys Research, National Institute of Public Health (INSP), Cuernavaca 62100, Mexico; 4 Genomics of Bone Metabolism Laboratory, National Institute of Genomic Medicine (INMEGEN), Mexico City 14610, Mexico; 5 Epidemiological and Health Services Research Unit, Morelos Delegation, Mexican Institute of Social Security, Cuernavaca 62000, Mexico; 6 Department of Health Policy and Management and UCLA-Kaiser Permanente Center for Health Equity, Fielding School of Public Health, University of California, Los Angeles, CA 90095, USA; 7 UCLA Center for Cancer Prevention and Control Research, Fielding School of Public Health, Los Angeles, CA 90095, USA; 8 Jonsson Comprehensive Cancer Center, Los Angeles, CA 90095, USA

**Keywords:** uric acid levels, bone mineral density, Mexican population

## Abstract

Background: Inconsistent epidemiological evidence between uric acid (UA) and bone mineral density (BMD) has been observed. Therefore, we evaluated the association between UA and BMD in Mexican adults. Methods: This analysis was conducted on 1423 participants from the Health Workers Cohort Study. We explored cross-sectional associations using linear regression and longitudinal associations using fixed-effects linear regression by sex and age groups (<45 and ≥45 years). Results: In females <45 years old, the cross-sectional analysis showed that UA levels were positively associated with total hip BMD. However, in the longitudinal analysis, we observed a negative association with the femoral neck and lumbar spine BMD. In contrast, in males <45 years old, we found an increase in total hip and femoral neck BMD in the groups with high levels of UA in the longitudinal association. On the other hand, in females ≥45 years old, we observed a longitudinal association between UA and loss of BMD at different sites. We did not observe an association between UA levels and BMD in males ≥45 years old. Conclusions: Our results suggest higher serum UA levels are associated with low BMD at different skeletal sites in Mexican females. Further studies are needed to delineate the underlying mechanisms behind this observation.

## 1. Introduction

Uric acid (UA) is an organic compound generated as the final product of purine metabolism [1]. UA has been considered an essential antioxidant [2]; it is a potent scavenger of free radicals and may contribute as an endogenous systemic antioxidant against bone deterioration [3,4]. Nevertheless, abnormally high levels could increase the risk of different diseases [5]. To date, the role of UA in bone mineral density (BMD) remains unclear. Different observational studies have demonstrated a positive association between UA and BMD (subjects with higher UA levels tend to have higher BMD). These findings have been observed at different skeletal sites such as the lumbar spine, hip, and femoral neck in both men and women, and most of the evidence has been obtained through cross-sectional studies [3,6,7,8]. However, recent studies have found a negative association between UA and BMD; a cross-sectional study showed that high levels of UA are a risk factor for vertebral fractures in postmenopausal women [9]. Longitudinal analysis showed that UA was negatively associated with BMD, and both studies were performed on subjects with type 2 diabetes (T2D) [9,10]. Nevertheless, other longitudinal studies did not find an association between UA and BMD changes in postmenopausal women [11] or between UA and incident fractures [12,13]. Furthermore, a mendelian randomization analysis in 241 elderly men and 1108 postmenopausal women did not show a causal association of UA with BMD [14]. Additionally, experiments using hyperuricemic and normouricemic rats reported no relationship between UA with BMD [15]. Therefore, we aim to evaluate the cross-sectional and longitudinal association between UA levels and BMD in Mexican adults.

## 2. Materials and Methods

### 2.1. Study Population

The data used in this study were derived from the Health Worker Cohort Study (HWCS), collected at baseline from 2004–2006 and through follow-up waves from 2010–2012 [16]. Workers and their relatives were invited by the Mexican Institute of Social Security (IMSS) of Cuernavaca Morelos. In this analysis, we included 1737 participants aged >20 with BMD measurement. Participants with kidney damage (n = 23), missing data on the food-frequency questionnaire (FFQ; those who answered <75% of the questionnaire or who had missing data in an entire section of the questionnaire; n = 99), or with implausible energy consumption estimated through a generalized extreme studentized deviate test (n = 49; <500 kcal/d or >6500 kcal/d) were excluded. We also excluded individuals with missing information on UA (n = 3), creatinine (n = 14), or smoking (n = 126). The final sample for this analysis consisted of 1423 participants (Supplementary Figure S1). The Research, Ethics, and Biosecurity Committee from the IMSS evaluated and accepted the study protocol (12CEI 09 006 14). Written informed consent was obtained from all the participants.

### 2.2. Bone Mineral Density Measurement

BMD measurements were obtained with a dual-energy X-ray absorptiometry (DEXA) Lunar DPX NT instrument (Lunar Radiation Corp., Madison, WI, USA) by trained examiners. The same machine was used for both study waves (2004–2006 and 2010–2012). Measurement sites were the femoral neck, total hip, and lumbar spine (L1–L4). Trained technicians performed daily quality control checks using the manufacturer phantom; the daily variation coefficient was within usual operational standards, and the in vivo variation coefficient was lower than 1.0–1.5% [16].

### 2.3. Uric Acid Level Assessment

At baseline and follow-up, fasting UA levels were determined through the enzymatic colorimetric method using a SYNCHRON CX system from Beckman Coulter [16]. We evaluated UA changes as a continuous as well as a categorical variable (categories defined by quartiles and hyperuricemia by serum concentrations UA cut-off points of ≥7.0 mg/dL in men and ≥5.7 mg/dL in women) [17].

### 2.4. Covariate Assessment

Demographic data such as age, sex, medication use (hormone replacement therapy [HRT]), consumption of calcium supplements, smoking, physical activity, and dietary intake were obtained from self-administered questionnaires in both waves [16].

Baseline age was categorized as <45 and ≥45 years old because, based on previous studies, endocrine changes affecting BMD in women occur around the age of 45 years [18,19]. Furthermore, we explored the age categories <50 and ≥50 years according to the WHO criteria for osteoporosis [20,21]. Smoking status was classified as never smokers, former smokers, and current smokers. Information on dietary intake was assessed using a 116-item semi-quantitative FFQ based on a previously validated FFQ [16] and using food composition tables compiled by the National Institute of Public Health [16]. Dietary inflammatory index (DII) scores were calculated using a method previously reported by Shivappa et al., with 30 of the 45 food parameters, such as total energy, carbohydrate, protein, total fat, cholesterol, saturated fatty acids (SFAs), monounsaturated fatty acids (MUFAs), polyunsaturated fatty acids (PUFAs), alcohol, fiber, caffeine, iron, magnesium, niacin, riboflavin, selenium, thiamine, beta carotene, zinc, folic acid, n-3 fatty acids, n-6 fatty acids, onion, trans fat, and vitamins A, C, D, E, B6, and B12 [22]. The leisure time physical activity (PA) was estimated with data from a previously validated PA questionnaire [23]. World Health Organization’s definition was used to establish two categories: inactive (<150 min/week of moderate to vigorous activity) or active (≥150 min/week of moderate to vigorous activity) [24]. T2D was defined as self-reported physician-diagnosed diabetes, use of hypoglycemic medication, or fasting glucose established cut-off points of >126.0 mg/dL [25].

Standing height and weight were measured with standardized procedures. We calculated participants’ body mass index (BMI) (kg/m^2^) at each wave, and the WHO’s cut-off points were used for the classification of BMI status [26,27]. Serum creatinine levels were determined using a compensated kinetic Jaffe assay that offers results traceable to the isotope dilution mass spectrometry reference method. Glomerular filtration rate (GFR) was calculated using the Chronic Kidney Disease Epidemiology Collaboration (CKD-EPI), and values were expressed as mL/min/1.73 m^2^.

### 2.5. Statistical Analyses

For descriptive purposes, continuous variables were summarized with means and standard deviations (SD) or median and 25th percentile (P25)–75th percentile (P75), and categorical variables using percentages by sex and age groups. For Table 1, the differences by sex at baseline were analyzed with Wilcoxon for continuous variables and the z-statistic for testing the equality of proportions for categorical variables. Differences between UA categories at baseline were analyzed with Dunn’s test for continuous variables and the z-statistic for testing the equality of proportions for categorical variables.

Differences between study stages were analyzed with the Wilcoxon rank-sum test for paired samples, the paired t-test for continuous variables, and McNemar’s test for categorical variables.

Cross-sectional associations between UA levels (continuous and categorical) and BMD were analyzed by linear regression models stratified by sex and age groups. The models were adjusted by age, BMI category (normal, overweight, and obesity, use of HRT, T2D, calcium supplement consumption, CKD-EPI equation, DII, energy-adjusted calcium intake, smoking, and leisure time PA. Longitudinal associations with UA levels continuously and categorically were analyzed by fixed-effects (FE) linear regression models stratified by sex and age groups. FE models analyze within-person change while eliminating time-invariant confounding (between-individual differences) [28]. The models were adjusted by covariates changing over time, such as BMI category (normal, overweight, and obese), use of HRT, T2D, CKD-EPI equation, DII, calcium supplements consumption, energy-adjusted calcium intake, smoking, and leisure time PA.

The level of statistical significance was set at α < 0.05. All statistical analyses were performed using Stata 14.2.

## 3. Results

### 3.1. Cross-Sectional Associations between UA and BMD Levels by Sex and Age Groups

We explored the characteristics by sex. Statistically significant differences were observed between females and males. Exposures such as amount overweight, UA, hyperuricemia, creatinine, DII, energy, smoking status, and phosphorous intake were higher in males than females. Additionally, females had higher body fat proportions, calcium supplements, and CKD-EPI than males. Furthermore, we observed that females had less hip BMD, femoral neck BMD, and lumbar spine BMD than males. We did not observe statistically significant differences in calcium intake by sex (Table 1). In addition, we explored the characteristics at baseline by UA categories. In females and males <45 years, statistically significant differences were observed in BMI, amount overweight, body fat proportion, hyperuricemia, DII, and calcium intake between the lowest and the highest quartile of UA. In addition, differences in obesity and low hip BMD were observed in females. In the ≥45 years old group, BMI, amount overweight, obesity, body fat proportion, and hyperuricemia showed significant differences between UA categories in females and males. Furthermore, differences in females were observed at the hip and femoral neck BMD, while in males they were found in CKD-EPI and MDRD equations and current smoking status (Supplementary Table S1).

**Figure 1:**
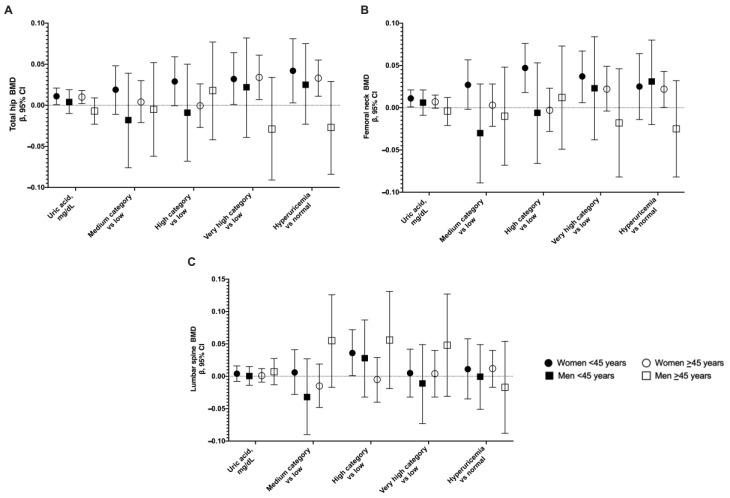
Cross-sectional association between UA and total hip BMD (**A**), femoral neck BMD (**B**), and lumbar spine BMD (**C**). Errors bars represent 95% confidence intervals.

Results from the linear regression showed a positive association between UA with hip and femoral neck BMD in females <45 years old. The adjusted model showed that an increase in 1 mg/dL of UA was associated with an increase in total hip BMD of 0.011 g/cm^2^ (95% CI 0.0009, 0.021). Furthermore, at hip BMD, this association was observed with hyperuricemia, and at the femoral neck, the association remained in the high and very high categories of UA (Figure 1 and Supplementary Table S2). In females ≥45 years old, we observed UA levels and hyperuricemia to be positively associated with hip BMD (β = 0.010, 95% CI 0.002, 0.018; and β = 0.033, 95% CI 0.011, 0.055; respectively). The association between UA and lumbar spine BMD was not significant in both age groups of females. We did not observe a cross-sectional association between UA and BMD in males in both age groups (Supplementary Tables S2 and S4).

### 3.2. Longitudinal Associations between UA and BMD Levels by Sex and Age Groups

The final analysis included 1423 participants with a mean time between baseline and second wave of 6.8 years (SD 1.4). Females made up 73.9% of the participants, of which 56.7% were ≥45 years old. At baseline, the median age of the group <45 years old was 36 years (P25-P75: 31–41) in females and males, while for the ≥45 years old group, the median age was 55 years (P25-P75: 49–61) for females and 54 years (P25-P75: 55–67) for males.

Measurements along the two-time points showed a significant increase of UA in females in both age groups and a decrease in total hip and lumbar spine BMD. Femoral neck BMD increased in <45 years and decreased in ≥45 years. In both age groups, hyperuricemia, BMI, body fat proportion, DII, and smoking status increased, while creatinine, energy, and calcium intake decreased. Furthermore, in females ≥45 years old, the HRT and the high leisure time physical activity decreased.

On the other hand, in both age groups of males, a significant increase in body fat proportion and a decrease in creatinine, energy calcium intake, hip BMD, and femoral neck BMD were observed. In addition, in <45 years, BMI, CKD-EPI equation, and smoking status increased. In ≥45 years, UA levels, hyperuricemia, lumbar spine BMD, and DII score increased (Table 2).

**Figure 2:**
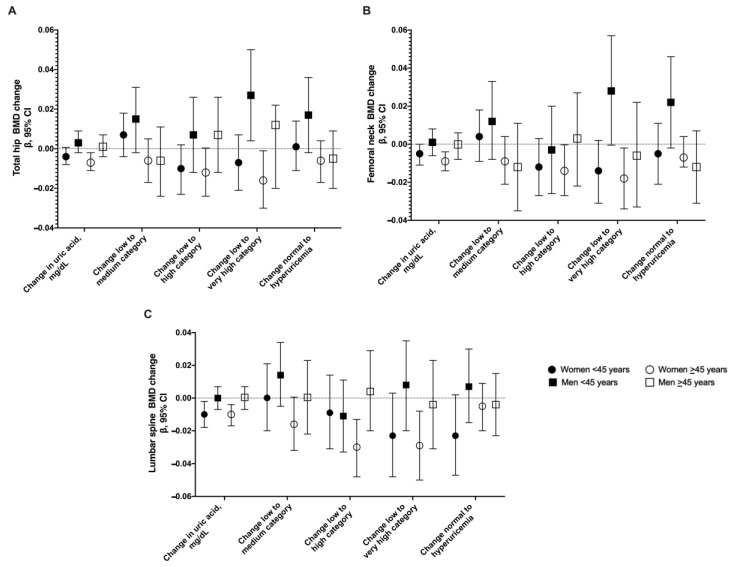
Longitudinal association between changes in UA and changes in total hip BMD (**A**), femoral neck BMD (**B**), and lumbar spine BMD (**C**). Errors bars represent 95% confidence intervals.

In females ≥45 years old, the fixed-effects linear regression showed that UA was negatively associated with hip, femoral neck, and lumbar spine BMD change (Figure 2 and Supplementary Table S3). The adjusted model showed that an increase in 1 mg/dL of UA was associated with a decrease in total hip BMD of 0.007 g/cm^2^ (95% CI −0.011, −0.002), in femoral neck −0.0009 g/cm^2^ (95% CI −0.014, −0.004), and in lumbar spine −0.010 g/cm^2^ (95% CI −0.017, −0.004). We observed that these findings were maintained when evaluating change from a low to a high category of UA and from a low to a very high category of UA for the different sites. In females <45 years old, significant associations of UA levels were observed for femoral neck and lumbar spine BMD (Figure 2 and Supplementary Table S3). When we stratified <50 and ≥50 years, the associations remained in the same direction but were not statistically significant at >50 years (Supplementary Table S5).

In males, we did not observe a significant association between UA and BMD in both age groups as a continuous variable. Nevertheless, in the evaluation by change of categories in <45 years old, we found a positive association; change from a low to a very high category of UA increased hip BMD by 0.027 g/cm^2^ (95% CI 0.004, 0.050). In addition, we also observed a change from normal to hyperuricemia increased femoral neck BMD in 0.022 g/cm^2^ (95% CI 0.002, 0.046) (Figure 2 and Supplementary Table S3). When we stratified <50 and ≥50 years, the associations were similar (Supplementary Table S5).

## 4. Discussion

Our results suggest a positive cross-sectional association between UA with hip and femoral neck BMD in females <45 years old. Nevertheless, a longitudinal negative association was observed between increased levels of UA and decreased hip, femoral neck, and lumbar spine BMD in Mexican women ≥45 years old. Meanwhile, in women <45 years old, this association was only present at the femoral neck and lumbar spine BMD.

It is essential to interpret the results of the cross-sectional analysis with caution due to potential residual confounding with unmeasured variables (shared genetic variants or environmental causes). In our cross-sectional analysis, we observed in women <45 years old a positive association between UA levels and total hip and femoral neck BMD. Estrogens could partially explain this observation; estrogens are one key regulator of bone metabolism, having direct effects on osteocytes (decreasing bone remodeling), osteoblasts (maintaining bone formation), and osteoclasts (decreasing bone resorption). Therefore, a decrease in BMD has been observed during menopause due to estrogen deficiency [29]. In previous studies, women with low estradiol levels (<11 pg/mL) had a more than twofold increased relative risk of fracture (Risk Ratio [RR] 2.2, 95% CI 1.2, 4.0) [30]. A longitudinal study of 1902 pre-or early perimenopausal women assessed bone loss at each transition stage due to a decline of ovarian function. Little change was observed during the pre-or early menopause stages in the lumbar spine and total hip BMD, but the bone loss was accelerated in late perimenopause (0.018 g/cm^2^ and 0.010 per year, respectively, *p* < 0.001) when adjusted for age, menopausal stage, weight, smoking, ethnicity, intake of vitamin D, calcium, and alcohol [31]. Estrogens have also been studied because of their effect on UA levels, due to a higher renal clearance of urate [32,33]. To our knowledge, there is no evidence evaluating the association between estrogen and UA levels with BMD. Additional studies are needed to determine the role of estrogen and uric acid levels on BMD in the Mexican population.

Several studies have shown a positive association between UA levels and BMD, especially in postmenopausal women. A large cross-sectional study of 7502 postmenopausal women found, after adjusting for multiple confounders (age, weight, height, GFR, calcium and phosphorus concentrations, and lifestyle factors), that serum UA levels were positively associated with BMD at all sites (per 1 mg/dL UA increase: lumbar spine β: 0.009 g/cm^2^, femoral neck β: 0.004 g/cm^2^, total hip β: 0.006 g/cm^2^, and trochanter β: 0.005 g/cm^2^, *p* < 0.001) [34]. Similarly, it was observed in a cross-sectional analysis with 356 twin women ≥45 years old that UA levels were positively associated with BMD in all skeletal sites (lumbar spine 0.190 g/cm^2^, femoral neck 0.169 g/cm^2^, total hip 0.167 g/cm^2^, total forearm 0.136 g/cm^2^, and whole body 0.170 g/cm^2^, *p* < 0.01), after adjusting for multiple confounders (age, GFR, calcium, CTX-I levels, FM/LM/Ht^2^, HRT, and lifestyle factors) [35].

In agreement with other studies, we observed higher UA levels in men than in women and across age categories in women. [36,37].The National Health and Nutrition Examination Survey (NHANES) 2015–2016 reported that men had higher UA levels (6.04 mg/dL) than women (4.79 mg/dL), and the hyperuricemia prevalence increased according to age categories; the highest was at >80 years (27.8%), followed by 26.1% (60–79 years) and 18.7% (40–59 years) [37]. Compared to older men, one explanation for the decreased UA levels in older women is the use of HRT due to its effect on insulin sensitivity, reducing UA concentrations by increasing its renal excretion [38].

On the other hand, some studies have shown that uric acid causes mitochondrial oxidative stress, stimulating fat accumulation independent of excessive caloric intake [39]. A meta-analysis found that for obesity (BMI > 30 kg/m^2^), the age-adjusted relative ratio of gout was 2.24 (95% CI 1.76–2.86) [40]. In our research, we found in men and women a higher prevalence of obesity in those participants with UA levels in the very high category compared to those in lower categories. However, the association between the two variables must be taken with caution because serum uric acid influenced obesity, most likely together with other related factors not analyzed in this study, such as diet.

For our longitudinal analysis, it is important to consider that the fixed-effects method controls for unchanging variables, such as genetic factors. While there is currently controversial information regarding the association between UA and BMD, few studies have demonstrated a negative association. A cross-sectional study, including 356 postmenopausal women and 512 men older than 50 diagnosed with T2D, found that higher UA levels were significantly associated with vertebral fractures only in women (odds ratio [OR] = 1.38, 95 %CI = 1.01–1.88, *p*-value = 0.041) [41]. In older adults from a Cardiovascular Health Study, men with higher urate levels had a 60% increased risk of hip fracture (Hazard Ratio [HR] 1.6; 95% CI 1.1, 2.5). In addition, it was observed that urate levels >10 mg/dL had a higher risk of hip fracture (HR 3.0, 95% CI 1.5, 6.1), followed by urate >9 mg/dL (HR 1.9, 95% CI 1.2, 3.0) and >8 mg/dL (HR 1.4; 95% CI 1.1, 1.8). In contrast, there was no association between UA levels and hip fractures in women [13]. One explanation for this negative association is the generation of oxidative stress by hyperuricemia, which inhibits bone cell differentiation and promotes osteoclast function [38,42,43]. Furthermore, during an acute gout attack, proinflammatory cytokines (interleukin-1, interleukin-6, interleukin-8, and Tumor Necrosis Factor-α) are released and promote osteoclast differentiation, increasing bone resorption [36].

There is little information regarding the association between UA and BMD in men, especially younger adults. In men, we did not observe an association between UA and BMD in the cross-sectional and longitudinal analysis while considering BMD as a continuous variable. Nevertheless, we found a positive association when category change was observed, from a low to a very high UA with increased hip BMD and from normal to hyperuricemia with femoral neck BMD. The evidence reinforces that the female sex hormones may be regulating UA handling machinery [33]. Estrogen could explain the observed sex differences in UA levels associated with BMD. Therefore, understanding the role of estrogen in regulating serum uric acid levels is critical to unraveling the complex mechanisms of UA and BMD.

In a cross-sectional study based on the NHANES from 1999 to 2006 with 6704 men >18 years, no statistically significant association between UA and lumbar spine BMD was observed after adjusting for confounding factors [44]. Similar results were observed in a study with 943 males and 4256 postmenopausal females; the association between UA levels and femoral neck, total hip, and L1-L4 BMD was observed in women, but not in men after adjusting for confounders [45]. A possible explanation could be the paradoxical antioxidant effects of UA acting as an electron donor and chelating metal ions to convert them into less reactive forms incapable of catalyzing free radical reactions, preventing osteoclast bone resorption [2]. However, studies in younger populations of men are needed to confirm these findings.

The potential causes for discrepancy between prior studies might be due to different population characteristics (genetic background, age, obesity, and body fat proportion) or differences in potential confounders adjusted, primarily related to lifestyle factors. In our case, we adjusted by DII, but in other studies, adjustments were not made for vegetables, alcohol, and calcium consumption.

The present study has some strengths and limitations. The main strength is the longitudinal design that includes women and men of a wide range of ages, allowing age stratification (<45 and ≥45 years). Standardized procedures with validated instruments and trained personnel were used to assess the clinical variables. The main limitations were the low prevalence of osteoporosis and the small number of women who used HTR; therefore, it was impossible to evaluate these associations and interactions. Unfortunately, we did not have information on menopausal status for all women; therefore, we used age categories as a proxy according to previous studies [18,19]. We have no information regarding the consumption of medications such as diphosphonate, glucocorticoids, and allopurinol that affect BMD or UA. However, the consumption of HRT and diuretics is low, so we consider that these could not affect the results.

## 5. Conclusions

In conclusion, our results suggest that higher serum UA levels are associated with low BMD at different skeletal sites in Mexican females. This effect on bone metabolism is independent of the confounding variables analyzed (BMI category, use of HRT, calcium supplement consumption, energy-adjusted calcium intake, smoking, and PA).

## Figures and Tables

**Table 1:** Baseline characteristics of the Health Workers Cohort Study (HWCS) by sex.

	Total n = 1423	Females n = 1051	Males n = 372
Age ^a^, years	46(37–55)	46(37–56)	45(36–54) *
BMI ^a^, kg/m^2^	26.1(23.6–29.0)	25.9(23.5–29.0)	26.5(24.2–28.9)
Overweight, %	43.1	40.8	49.5 **
Obesity, %	19.3	19.6	18.6
Body fat proportion ^a^	40.7(33.8–45.8)	43.2(38.7–47.2)	30.6(27.0–35.0) ***
Diabetes, %	8.6	8.7	8.3
Uric acid ^a^, mg/dL	4.8(3.9–5.9)	4.4(3.7–5.3)	6.0(5.2–6.8) ***
Hyperuricemia, %	18.2	16.9	21.8 *
Creatinine ^a^, mg/dL	0.9(0.8–1)	0.8(0.7–1.0)	1.0(0.9–1.2) ***
CKD-EPI equation ^a^	98.1(91.1–105.5)	98.1(90.8–105.9)	97.8(91.6–104.7)
Dietary inflammatory index ^a^	0.09(−1.41, 1.85)	−0.05(−1.48, 1.78)	0.37(−1.09, 2.25) **
Energy ^a^, kcal/day	1980(1528–2536)	1928(1512–2451)	2150(1612–2785) ***
Smoking status, %			
Past, %	28.3	23.8	41.1 ***
Current, %	16.0	13.7	22.3 ***
Phosphorous intake ^a^, mg/day	1326(1019–1742)	1298(999–1689)	1419(1085–1835) **
Calcium intake ^a^, mg/day	985(817–1203)	993(825–1208)	950(794–1196)
Calcium supplements, %	14.3	17.9	4.3 ***
Hormone replacement therapy, %	-	6.1	-
Hip BMD ^a^, g/cm^2^	1.019(0.923–1.112)	0.996(0.904–1.082)	1.084(0.997–1.189) ***
Hip T-score ^a^	−0.10(−0.82, 0.61)	−0.09(−0.85, 0.61)	−0.11(−0.72, 0.64)
Low hip BMD, %	20.2	21.0	17.7
Femoral neck BMD ^a^, g/cm^2^	0.978(0.884–1.077)	0.960(0.867, 1.050)	1.029(0.942, 1.145) ***
Femoral neck T-score ^a^	−0.58(−1.35, 0.22)	−0.69(−1.45, 0.09)	−0.34(−0.95, 0.58) ***
Lumbar spine BMD ^a^, g/cm^2^	1.113(1.009–1.223)	1.102(0.999–1.213)	1.148(1.041–1.258) ***
Lumbar spine T-score ^a^	−0.78(−1.63, 0.09)	−0.78(−1.63, 0.09)	−0.77(−1.64, 0.09)
High leisure time physical activity, %	38.5	35.3	47.4 ***

^a^ Median (P25-P75). Hyperuricemia was defined as 7.0 mg/dL among males and 5.7 mg/dL among females. The *p*-values of the statistical tests were calculated using the Wilcoxon test for continuous variables and the tests on the equality of proportions for categorical variables. * *p* < 0.05, ** *p* < 0.01, *** *p* < 0.001.

**Table 2:** Baseline and follow-up characteristics of the Health Workers Cohort Study (HWCS) by sex and age groups (n = 1423).

	<45 Years				≥45 Years			
	Females (n = 455)		Males (n = 181)		Females (n = 596)		Males (n = 191)	
	Baseline	Follow-Up	Baseline	Follow-Up	Baseline	Follow-up	Baseline	Follow-Up
Age ^a^, years	36 (31–41)	43 (38–48) ***	36 (31–40)	43 (38–47) ***	55 (49–61)	61 (56–68) ***	54 (48–60)	60 (55–67) ***
BMI ^a^, kg/m^2^	24.7 (22.5–27.8)	25.7 (23.3–29.2) ***	26.2 (24.2–28.8)	27.9 (24.7–29.5) ***	26.8 (24.5–29.9)	26.9 (24.6–30.0) *	26.7 (24.5–29.0)	26.9 (24.0–29.1)
Overweight, %	33.0	34.4	51.4	49.7	46.7	47.0	49.5	47.5
Obesity, %	13.6	19.9*	18.2	21.0	24.1	24.9	18.6	186
Body fat proportion ^a^	40.8 (40.2–41.4)	44.0 (43.4–44.6) ***	31.1 (29.6–32.5)	32.3 (31.3–33.2) *	43.9 (43.4–44.4)	45.0 (44.5–45.5) ***	30.9 (30.0–31.8)	31.9 (31.0–32.8) **
Uric acid ^a^, mg/dL	4.1 (4.0–4.2)	4.4 (4.3–4.5) ***	6.4 (6.2–6.6)	6.3 (6.1–6.5)	4.9 (4.8–5.0)	5.2 (5.1–5.3) ***	5.9 (5.7–6.1)	6.1 (5.9–6.3) *
Hyperuricemia, %	8.5 (6.0–11.1)	12.7 (9.6–15.8) *	26.0 (19.5–324)	24.9 (18.5–31.2)	23.8 (20.4–27.2)	30.0 (26.4–33.6) **	17.5 (12.1–22.9)	25.3 (19.0–31.4) *
Creatinine ^a^, mg/dL	0.84 (0.83–0.86)	0.72 (0.71–0.73) ***	1.03 (1.01–1.06)	0.90 (0.87–0.92) ***	0.84 (0.83–0.86)	0.72 (0.71–0.74) ***	1.04 (1.01–1.06)	0.93 (0.90–0.96) ***
CKD-EPI equation ^a^	106.6 (105.8–107.4)	106.6 (105.8–107.4)	104.7 (103.5–105.9)	106.2 (104.8–107.6) *	92.4 (91.7–93.1)	92.6 (91.8–93.3)	92.0 (90.2–93.9)	91.4 (90.0–92.8)
Dietary inflammatory index ^a^	0.11 (−1.43, 1.84)	0.70 (−1.09, 2.36) **	0.63 (−1.03, 2.40)	0.90 (−0.68, 2.50)	−0.12 (−1.57, 1.66)	0.50 (−0.90, 2.14) ***	0.25 (−1.11, 2.02)	0.81 (−0.75, 2.38) *
Energy ^a^, kcal/day	2148 (2068–2227)	1880 (1806–1953) ***	2310 (2177–2442)	2122 (1985–2261) **	2008 (1944–2073)	1750 (1694–1807) ***	2224 (2103–2346)	1908 (1803–2013) ***
Smoking status, %								
Past, %	21.2	26.9 *	34.8	47.5 *	25.9	32.5 *	46.4	55.7
Current, %	14.9	12.7	25.4	18.8	13.0	7.9 **	19.1	12.4
Phosphorous intake ^a^, mg/day	1308 (1015–1731)	1070 (766–1451) ***	1504 (1126–1838)	1190 (944–1621) ***	1294 (994–1681)	1045 (786–1434) ***	1378 (1037–1815)	1089 (794–1491) ***
Calcium intake ^a^, mg/day	1062 (1009–1113)	867 (820–914) ***	1001 (927–1075)	894 (814–975) *	1059 (1013–1105)	867 (828–906) ***	1043 (961–1126)	842 (774–909) ***
Calcium supplements, %	7.0	6.8	1.1	0.0	26.2	26.1	7.7	0.0 ***
Hormone replacement therapy, %	3.3	3.7	-	-	8.4	5.4 *	-	-
Diuretics, %	0.9	1.5	0	0.6	1.2	2.9	0.5	3.1
Hip BMD ^a^, g/cm^2^	1.040 (1.029–1.051)	1.027 (1.015–1.038) ***	1.126 (1.104–1.147)	1.111 (1.089–1.132) **	0.962 (−0.051, −0.044)	0.914 (0.904, 0.925) ***	1.061 (1.039–1.082)	1.037 (1.015–1.058) ***
Hip T-score ^a^	0.20 (−0.37, 0.90)	0.06 (−0.57, 0.81) ***	0.16 (−0.49, 0.86)	0.09 (−0.65, 0.66) ***	−0.40 (−1.11, 0.41)	−0.75 (−1.44, −0.09) ***	−0.32 (−0.95, 0.34)	−0.47 (−1.11, 0.27) ***
Low hip BMD, %	9.8	12.9	11.6	14.4	29.8	41.3 ***	31.4	23.7
Femoral neck BMD ^a^, g/cm^2^	1.008 (0.996–1.019)	1.019 (1.008–1.030) ***	1.096 (1.073, 1.118)	1.073 (1.051–1.095) ***	0.917 (0.907–0.928)	0.868 (0.858–0.877) ***	0.994 (0.973–1.026)	0.962 (0.941–0.983) ***
Femoral neck T-score ^a^	−0.19 (−0.86, 0.55)	−0.30 (−0.84, 0.35)	0.11 (−0.67, 0.93)	−0.03 (−0.75, 0.60)	−1.03 (−1.74, −0.24)	−1.25 (−1.84, −0.61)	−0.63 (−1.29, 0.06)	−0.86 (−1.46, −0.15)
Lumbar spine BMD ^a^, g/cm^2^	1.172 (1.159–1.184)	1.159 (1.147–1.172) **	1.162 (1.142–1.183)	1.162 (1.140–1.184)	1.045 (1.032–1.058)	1.006 (0.994–1.018) ***	1.152 (1.127–1.178)	1.167 (1.142–1.193) **
Lumbar spine T-score ^a^	−0.26 (−0.88, 0.45)	−0.41 (−1.08, 0.44) ***	−0, 70 (−1.45, 0.16)	−0.76 (−1.45, 0.12)	−1.33 (−2.15, −0.40)	−1.70 (−2.42, −0.78) ***	−0.88 (−1.80, 0.09)	−0.77 (−1.71, 0.21) *
High leisure time physical activity, %	30.4	32.0	47.8	39.2	38.9	32.0 *	46.9	40.7

^a^ Median (P25-P75). The *p*-values of the statistical tests were calculated using the Wilcoxon rank-sum test (for paired samples) for continuous variables and McNemar’s test for categorical variables. * *p* < 0.05, ** *p* < 0.01, *** *p* < 0.001.

## Data Availability

The datasets analyzed in this study are available from the corresponding author upon reasonable request.

## Supplementary Materials

The following supporting information can be downloaded at: https://www.mdpi.com/article/10.3390/nu14204245/s1, Figure S1: Flowchart of study population; Table S1: Baseline characteristics of the Health Workers Cohort Study (HWCS) by uric acid categories (n = 1423); Table S2: Cross-sectional association between uric acid and BMD at baseline by sex and age groups; Table S3: BMD change according to changes in uric acid between baseline and follow-up by sex and age groups; Table S4: Cross-sectional association between uric acid and BMD at baseline by sex and age groups (<50 and ≥50 years old); Table S5: BMD change according to changes in uric acid between baseline and follow-up by sex and age groups (<50 and ≥50 years old).
