# Supplementary material for: Uric Acid Levels Are Associated with Bone Mineral Density in Mexican Populations: A Longitudinal Study
Source: Nutrients. 2022 Oct 12;14(20):4245. doi: 10.3390/nu14204245 (PMC9612067; doi:10.3390/nu14204245)
Supplement: Supplementary file 1 [file nutrients-14-04245-s001.zip › nutrients-1917908-supplementary.pdf]

Supplementary Figure S1 Flowchart of study population

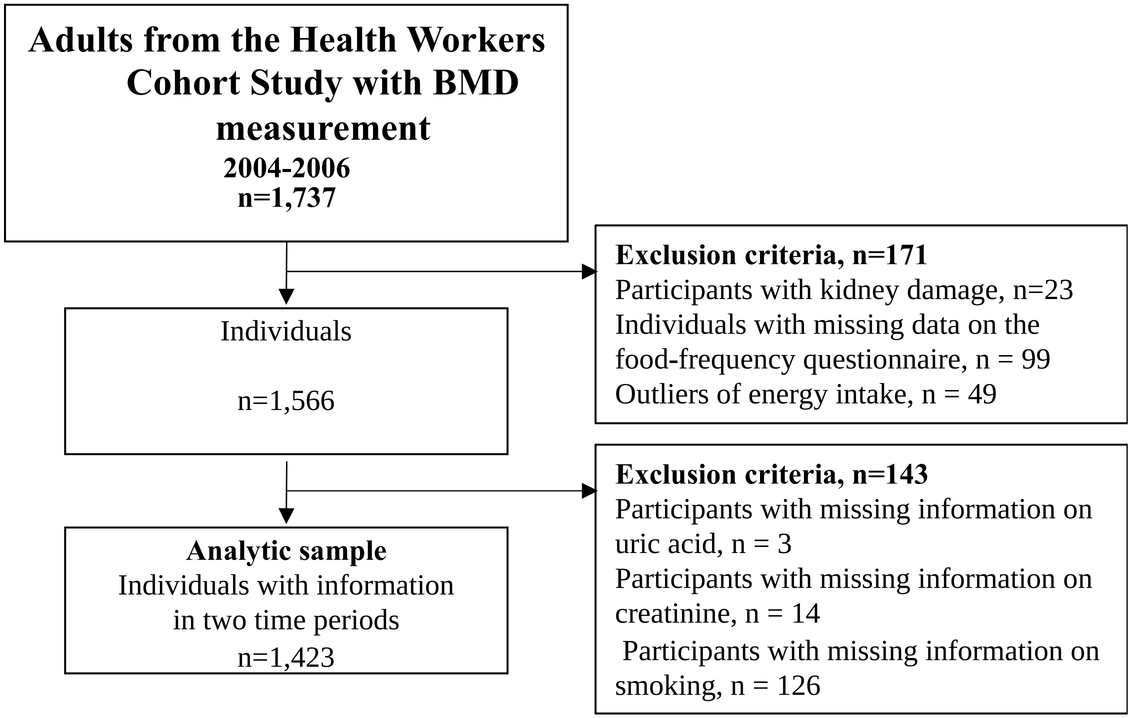

Supplementary Table S1. Baseline characteristics of the Health Workers Cohort Study (HWCS) by uric acid categories (n=1,423)

	<45 years				≥45 years			
	Females (n=455) Uric acid categories at baseline[a]		Males (n=181) Uric acid categories at baseline[a]		Females (n=596) Uric acid categories at baseline[a]		Males (n=191) Uric acid categories at baseline[a]	
	Low n=116	Very high n=111	Low n=48	Very high n=44	Low n=150	Very high n=147	Low n=51	Very high n=48
Age[b], years	35 (30-39)	38 (33-42)**	36 (32-40)	37 (32-40)	50 (47-57)	56 (52-63)***	52 (48-59)	54 (50-61)
BMI[b], kg/m²	23.1 (21.3-25.5)	26.7 (23.5-29.6)***	25.9 (23.0-28.5)	27.7 (26.1-29.7)**	25.6 (23.6-28.4)	28.4 (26.3-31.9)***	25.2 (24.0-26.9)	27.8 (25.7-31.4)**
Overweight, %	26.5	43.2**	41.7	63.6*	41.5	50.3	43.1	43.8
Obesity, %	4.3	24.3***	18.8	20.5	16.5	35.2***	7.8	33.3**
Body fat proportion[b]	38.6 (33.3-42.9)	43.6 (40.0-48.2)**	29.3 (24.5-32.9)	33.6 (29.8-38.3)**	42.4 (37.4-45.7)	44.7 (40.1-49.8)***	29.9 (25.8-32.4)	32.1 (29.5-37.0)***
Diabetes, %	1.7	6.3	6.3	2.3	10.7	16.6	19.6	6.4
Uric acid[b], mg/dL	2.8 (2.4-3.0)	5.5 (5.1-6.0)***	4.9 (4.6-5.2)	7.9 (7.4-8.9)***	3.6 (3.2-3.8)	6.3 (6.0-7.0)***	4.6 (4.2-4.8)	6.4 (6.1-6.5)***
Hyperuricemia, %	0.0	35.1***	0.0	100.0***	0.0	100.0***	0.0	70.8***
Creatinine[b], mg/dL	0.9 (0.8-0.9)	0.8 (0.7-1.0)	1.0 (0.9-1.1)	1.0 (0.9-1.2)	0.9 (0.7-1.0)	0.8 (0.7-1.0)	1.0 (0.9-1.2)	1.1 (0.9-1.2)
CKD-EPI equation[b]	105.9 (101.2-110.8)	105.5 (97.7-113.8)	104.1 (101.9-109.4)	103.4 (97.3-109.9)	93.7 (89.0-98.3)	90.3 (82.8-95.7)	91.5 (85.9-97.3)	90.1 (82.6-96.0)***
Dietary inflammatory index[b]	0.04 (-1.43,1.69)	-0.02 (-1.22,1.83)	0.11 (-1.43,1.78)	1.82 (-0.31,2.82)**	-0.42 (-2.09,1.58)	0.03 (-1.20,1.58)	0.18 (-1.72,1.81)	0.59 (-1.51,2.40)
Energy[b], kcal/day	2062 (1562-2533)	2036 (1552-2575)	2341 (1714-3077)	1781 (1467-2656)	1957 (1524,2466)	1903 (1465-2412)	2158 (1573-2654)	2175 (1487-2858)
Smoking status, %								
Past, %	21.4	27.0	29.2	27.3	21.7	22.8	54.9	40.0
Current, %	12.8	17.1	31.3	29.6	14.5	12.4	11.8	35.4**

Phosphorous intake[b], mg/day	1355 (1072,1676)	1311 (1041,1762)	1652 (1226,1998)	1250 (968,1742)*	1456 (954,1738)	1254 (998,1638)	1450 (1145,1920)	1285 (972,1629)
Calcium intake[b], mg/day	953 (724-1261)	916 (706-1336)***	1085 (739-1460)	744 (533-1021)**	1006 (664-1399)	875 (664-1247)	1058 (682-1428)	769 (609-1057)
Calcium supplements, %	7.7	8.1	0.0	0.0	24.3	24.8	7.8	10.4
Hormone replacement therapy, %	2.3	2.7	-	-	5.9	6.9	-	-
Hip BMD[b], g/cm[2]	1.001 (0.948-1.072)	1.065 (0.974-1.153)	1.125 (1.032-1.237)	1.178 (1.060-1.246)	0.961 (0.873-1.069)	0.990 (0.893-1.081)***	1.046 (0.962-1.112)	1.047 (0.947-1.119)
Hip T-score[b]	0.003 (-0.48,0.55)	0.46 (-0.27,1.19)***	0.17 (-0.48,0.94)	0.53 (-0.28,1.01)	-0.39 (-1.12,0.52)	-0.08 (-0.84,0.64)*	-0.38 (-0.96,0.09)	-0.33 (-1.02,0.14)
Low hip BMD, %	15.4	5.4*	16.7	4.6	31.6	24.1	23.5	27.1
Femoral neck BMD[b], g/cm[2]	0.979 (0.902-1.057)	1.029 (0.942-1.119)	1.103 (0.975-1.193)	1.118 (1.018-1.237)	0.917 (0.840-1.015)	0.931 (0.825-1.013)***	0.983 (0.906-1.077)	0.986 (0.891-1.039)
Femoral neck T-score[b]	-0.70 (-1.20,0.17)	-0.03 (-0.74,0.71)***	0.08 (-0.73,0.78)	0.58 (-0.32,1.29)*	-1.01 (-1.65,-0.13)	-0.81 (-1.71,-0.18)	-0.63 (-1.23,0.03)	-0.63 (-1.35,-0.22)
Lumbar spine BMD[b], g/cm[2]	1.146 (1.075-1.238)	1.162 (1.089-1.240)	1.190 (1.051-1.278)	1.133 (1.040-1.249)	1.043 (0.965-1.203)	1.031 (0.929-1.136)	1.104 (0.974-1.223)	1.138 (1.039-1.229)
Lumbar spine T-score[b]	-0.33 (-0.97,0.34)	-0.29 (-0.89,0.25)	-0.41 (-1.59,0.46)	-0.76 (-1.60,0.09)	-1.24 (-1.90,0.01)	-1.42 (-2.24,-0.48)*	-1.14 (-2.16,-0.08)	-0.83 (-1.62,0.03)
High leisure time physical activity, %	32.5	26.1	47.9	53.5	44.1	37.9	45.1	43.8

[b]Median (P25-P75). Hyperuricemia was defined as 7.0 mg/dl among males and 5.7 mg/dl among females. The *p* values of the statistical tests were calculated using the Dunn ' s test for continuous variables and the tests on the equality of proportions for categorical variables. * p<0.05, ** p<0.01, *** p<0.001.

Supplementary Table S2. Cross-sectional association between uric acid and BMD at baseline by sex and age groups

	<45 years		≥45 years	
	**Females (n=455)** β (% 95 CI)	**Males (n=181)** β (% 95 CI)	**Females (n=596)** β (% 95 CI)	**Males (n=191)** β (% 95 CI)
	**Hip BMD**			
**Model 1**				
**Uric acid, mg/dL**	0.022(0.012,0.032)***	0.011(-0.003,0.025)	0.006(-0.003,0.016)	0.005(-0.010,0.020)
Low[a]	0.0	0.0	0.0	0.0
Medium category[a]	0.028(-0.003,0.059)	-0.017(-0.075,0.040)	-0.009(-0.039,0.021)	0.034(-0.026,0.094)
High category[a]	0.044(0.013,0.075)	-0.003(-0.062,0.057)	-0.013(-0.044,0.018)	0.049(-0.017,0.106)
Very high category[a]	0.065(0.033,0.100)***	0.023(-0.038,0.083)	0.027(-0.005,0.058)	0.012(-0.049,0.072)
Normal	0.0	0.0	0.0	0.0
Hyperuricemia	0.078(0.038,0.117)***	0.047(-0.002,0.095)	0.026(0.003,0.050)*	-0.005(-0.061,0.052)
**Model 2**				
**Uric acid, mg/dL**	0.012(0.002,0.021)*	0.005(-0.009,0.019)	0.010(0.002,0.018)*	-0.006(-0.021,0.010)
Low[a]	0.0	0.0	0.0	0.0
Medium category[a]	0.018(-0.011,0.048)	-0.023(-0.079,0.033)	0.003(-0.022,0.029)	0.003(-0.056,0.057)
High category[a]	0.028(-0.002,0.058)	-0.006(-0.064,0.051)	-0.0009(-0.026,0.025)	0.021(-0.037,0.080)
Very high category[a]	0.034(0.003,0.065)*	0.022(-0.035,0.080)	0.034(0.007,0.061)*	-0.020(-0.082,0.041)
Normal	0.0	0.0	0.0	0.0
Hyperuricemia	0.049(0.010,0.087)*	0.026(-0.020,0.075)	0.034(0.012,0.056)*	-0.026(-0.082,0.030)
**Model 3**				
**Uric acid, mg/dL**	0.011(0.0009,0.021)*	0.004(-0.010,0.019)	0.010(0.002,0.018)*	-0.007(-0.023,0.009)
Low[a]	0.0	0.0	0.0	0.0
Medium category[a]	0.019(-0.011,0.048)	-0.018(-0.076,0.039)	0.004(-0.021,0.030)	-0.005(-0.062,0.052)

High category[a]	0.029(-0.0005,0.059)	-0.009(-0.068,0.050)	-0.0005(-0.027,0.026)	0.018(-0.042,0.077)
Very high category[a]	0.032(0.001,0.064)*	0.022(-0.039,0.082)	0.034(0.007,0.061)*	-0.029(-0.091,0.034)
Normal	0.0	0.0	0.0	0.0
Hyperuricemia	0.042(0.003,0.081)*	0.025(-0.023,0.075)	0.033(0.011,0.055)*	-0.027(-0.084,0.029)
		**Femoral neck BMD**		
**Model 1**				
**Uric acid, mg/dL**	0.020(0.011,0.030)***	0.013(-0.001,0.028)	0.00006(-0.009,0.009)	0.004(-0.011,0.019)
Low[a]	0.0	0.0	0.0	0.0
Medium category[a]	0.034(0.003,0.064)*	-0.041(-0.102,0.020)	-0.015(-0.044,0.014)	0.026(-0.034,0.086)
High category[a]	0.058(0.028,0.088)***	0.002(-0.060,0.063)	-0.020(-0.050,0.010)	0.035(-0.026,0.097)
Very high category[a]	0.062(0.031,0.092)***	0.042(-0.020,0.103)	0.004(-0.027,0.034)	0.011(-0.050,0.071)
Normal	0.0	0.0	0.0	0.0
Hyperuricemia	0.059(0.021,0.098)**	0.052(0.002,0.102)*	0.015(-0.010,0.040)	-0.012(-0.068,0.045)
**Model 2**				
**Uric acid, mg/dL**	0.012(0.002,0.022)*	0.008(-0.006,0.023)	0.007(-0.001,0.015)	-0.003(-0.019,0.014)
Low[a]	0.0	0.0	0.0	0.0
Medium category[a]	0.026(-0.003,0.056)	-0.035(-0.092,0.022)	0.002(-0.023,0.027)	-0.003(-0.061,0.055)
High category[a]	0.045(0.016,0.074)**	0.001(-0.057,0.060)	-0.003(-0.029,0.022)	0.014(-0.046,0.073)
Very high category[a]	0.038(0.004,0.069)*	0.029(-0.030,0.088)	0.022(-0.005,0.049)	-0.008(-0.071,0.055)
Normal	0.0	0.0	0.0	0.0
Hyperuricemia	0.033(-0.005,0.071)	0.037(-0.012,0.085)	0.022(0.0004,0.043)*	-0.023(-0.080,0.033)
**Model 3**				
**Uric acid, mg/dL**	0.011(0.001,0.021)*	0.006(-0.009,0.021)	0.007(-0.0006,0.015)	-0.004(-0.021,0.012)
Low[a]	0.0	0.0	0.0	0.0
Medium category[a]	0.027(-0.002,0.0566)	-0.030(-0.089,0.028)	0.003(-0.022,0.028)	-0.010(-0.068,0.048)
High category[a]	0.047(0.018,0.076)**	-0.006(-0.066,0.053)	-0.003(-0.028,0.023)	0.012(-0.049,0.073)

Very high category[a]	0.037(0.006,0.067)*	0.023(-0.038,0.084)	0.022(-0.004,0.049)	-0.018(-0.082,0.046)
Normal	0.0	0.0	0.0	0.0
Hyperuricemia	0.025(-0.014,0.064)	0.031(-0.020,0.080)	0.022(0.0004,0.043)*	-0.025(-0.082,0.032)
		**Lumbar spine**		
**Model 1**				
**Uric acid, mg/dL**	0.006(-0.005,0.017)	0.002(-0.011,0.016)	-0.009(-0.020,0.002)	0.012(-0.007,0.030)
Low[a]	0.0	0.0	0.0	0.0
Medium category[a]	0.011(-0.0236,0.046)	-0.031(-0.088,0.025)	-0.041(-0.077,-0.005)	0.071(0.001,0.142)*
High category[a]	0.038(0.004,0.073)	0.025(-0.032,0.082)	-0.031(-0.068,0.006)	0.071(-0.001,0.144)
Very high category[a]	0.011(-0.024,0.047)	-0.011(-0.068,0.046)	-0.027(-0.065,0.010)	0.060(-0.012,0.131)
Normal	0.0	0.0	0.0	0.0
Hyperuricemia	0.019(-0.025,0.063)	0.002(-0.044,0.049)	-0.003(-0.034,0.028)	-0.004(-0.071,0.063)
**Model 2**				
**Uric acid, mg/dL**	0.005(-0.007,0.017)	0.0003(-0.014,0.014)	0.0008(-0.010,0.011)	0.009(-0.011,0.029)
Low[a]	0.0	0.0	0.0	0.0
Medium category[a]	0.006(-0.029,0.040)	-0.029(-0.086,0.027)	-0.016(-0.051,0.016)	0.057(-0.014,0.128)
High category[a]	0.035(0.0004,0.071)*	0.030(-0.028,0.088)	-0.007(-0.041,0.027)	0.066(-0.007,0.139)
Very high category[a]	0.006(-0.030,0.043)	-0.014(-0.072,0.045)	0.003(-0.033,0.039)	0.053(-0.025,0.131)
Normal	0.0	0.0	0.0	0.0
Hyperuricemia	-0.002(-0.029,0.062)	-0.003(-0.051,0.046)	0.012(-0.017,0.041)	-0.017(-0.088,0.053)
**Model 3**				
**Uric acid, mg/dL**	0.004(-0.008,0.016)	0.0004(-0.014,0.015)	0.001(-0.009,0.012)	0.007(-0.013,0.028)
Low[a]	0.0	0.0	0.0	0.0
Medium category[a]	0.006(-0.028,0.041)	-0.032(-0.090,0.027)	-0.015(-0.048,0.019)	0.055(-0.017,0.126)
High category[a]	0.036(0.001,0.072)*	0.028(-0.032,0.087)	-0.005(-0.040,0.029)	0.056(-0.019,0.131)
Very high category[a]	0.005(-0.032,0.042)	-0.011(-0.073,0.049)	0.004(-0.032,0.040)	0.048(-0.031,0.127)
Normal	0.0	0.0	0.0	0.0
Hyperuricemia	0.011(-0.035,0.058)	-0.0006(-0.051,0.049)	0.012(-0.017,0.040)	-0.017(-0.088,0.054)

[a] Categories defined by quartiles. Hyperuricemia was defined as 7.0 mg/dl among males and 5.7 mg/dl among females. * $p<0.05$, ** $p<0.01$, *** $p<0.001$. Model 1: unadjusted. Model 2: Adjusted by age, body mass index categories, smoking status, leisure time physical activity, hormone replacement therapy, CKD-EPI equation and diabetes. Model 3: included the additional predictors from model 2 plus adjustment for calcium intake, calcium supplements and dietary inflammatory index.

Supplementary Table S3. BMD change according to Changes in uric acid between baseline and follow-up by sex and age groups

	<45 years		≥45 years	
	**Females (n=455)**	**Males (n=181)**	**Females (n=596)**	**Males (n=191)**
	**β (% 95 CI)**	**β (% 95 CI)**	**β (% 95 CI)**	**β (% 95 CI)**
	**Hip BMD**			
**Model 1**				
**Uric acid, mg/dL**	-0.003 (-0.008,0.001)	0.039 (-0.002,0.010)	-0.009 (-0.013,-0.004)**	0.003 (-0.003,0.008)
Change low to medium category[a]	0.009 (-0.002,0.021)	0.013 (-0.004,0.029)	-0.007 (-0.019,0.005)	0.006 (-0.012,0.024)
Change low to high category[a]	-0.008 (-0.020,0.005)	0.004 (-0.014,0.022)	-0.016 (-0.029,-0.004)*	0.014 (-0.006,0.033)
Change low to very high category[a]	-0.003 (-0.016,0.011)	0.027 (0.004,0.050)*	-0.019 (-0.034,-0.005)*	0.012 (-0.009,0.033)
Change normal to hyperuricemia	-0.001 (-0.015,0.012)	0.017 (-0.002,0.037)	-0.007 (-0.018,0.003)	-0.002 (-0.017,0.014)
**Model 2**				
**Uric acid, mg/dL**	-0.005 (-0.009,-0.0001)*	0.003 (-0.002,0.009)	-0.008 (-0.012,-0.003)**	0.0002 (-0.005,0.005)
Change low to medium category[a]	0.007 (-0.004,0.018)	0.011 (-0.005,0.027)	-0.008 (-0.019,0.004)	-0.007 (-0.025,0.010)
Change low to high category[a]	-0.011 (-0.024,0.001)	0.004 (-0.014,0.022)	-0.014 (-0.026,-0.001)*	0.004 (-0.014,0.023)
Change low to very high category[a]	-0.009 (-0.022,0.005)	0.027 (0.004,0.049)*	-0.017 (-0.032,-0.003)*	-0.001 (-0.022,0.020)
Change normal to hyperuricemia	0.0004 (-0.013,0.014)	0.019 (-0.0005,0.038)	-0.007 (-0.017,0.004)	-0.007 (-0.021,0.007)
**Model 3**				
**Uric acid, mg/dL**	-0.004	0.003	-0.007	0.001

	(-0.008,0.0007)	(-0.002,0.009)	(-0.011,-0.002)**	(-0.004,0.007)
Change low to medium category[a]	0.007 (-0.004,0.018)	0.015 (-0.002,0.031)	-0.006 (-0.017,0.005)	-0.006 (-0.024,0.011)
Change low to high category[a]	-0.010 (-0.023,0.002)	0.007 (-0.012,0.026)	-0.012 (-0.024,0.0004)	0.007 (-0.012,0.026)
Change low to very high category[a]	-0.007 (-0.021,0.007)	0.027 (0.004,0.050)*	-0.016 (-0.030,-0.001)*	0.012 (-0.020,0.022)
Change normal to hyperuricemia	0.001 (-0.011,0.014)	0.017 (-0.002,0.036)	-0.006 (-0.017,0.004)	-0.005 (-0.020,0.009)
		**Femoral neck BMD**		
**Model 1**				
**Uric acid, mg/dL**	-0.006 (-0.011,-0.0003)*	0.002 (-0.006,0.009)	-0.009 (-0.014,-0.004)***	-0.0008 (-0.008,0.006)
Change low to medium category[a]	0.006 (-0.008,0.019)	0.006 (-0.014,0.027)	-0.008 (-0.020,0.005)	-0.001 (-0.024,0.022)
Change low to high category[a]	-0.011 (-0.026,0.004)	-0.011 (-0.034,0.012)	-0.015 (-0.019,-0.003)*	0.005 (-0.019,0.030)
Change low to very high category[a]	-0.014 (-0.030,0.003)	0.025 (-0.003,0.054)	-0.019 (-0.036,-0.006)*	0.004 (-0.023,0.031)
Change normal to hyperuricemia	-0.009 (-0.025,0.007)	0.022 (-0.003,0.047)	-0.010 (-0.021,0.002)	-0.012 (-0.031,0.007)
**Model 2**				
**Uric acid, mg/dL**	-0.006 (-0.011,-0.0008)*	0.0005 (-0.007,0.008)	-0.010 (-0.014,-0.005)***	-0.003 (-0.010,0.004)
Change low to medium category[a]	0.005 (-0.009,0.018)	0.005 (-0.015,0.025)	-0.010 (-0.023,0.002)	-0.013 (-0.037,0.010)
Change low to high category[a]	-0.013 (-0.028,0.002)	-0.010 (-0.033,0.013)	-0.016 (-0.030,-0.003)*	-0.004 (-0.028,0.021)
Change low to very high category[a]	-0.017 (-0.033,0.001)	0.025 (-0.004,0.053)*	-0.021 (-0.037,-0.005)*	-0.011 (-0.038,0.017)
Change normal to hyperuricemia	-0.006	0.024	-0.008	-0.016

	(-0.022,0.010)	(-0.0007,0.048)*	(-0.019,0.004)	(-0.035,0.003)
**Model 3**				
**Uric acid, mg/dL**	-0.005 (-0.011,0.000001)	0.001 (-0.006,0.008)	-0.0009 (-0.014,-0.004)	-0.001 (-0.008,0.006)
Change low to medium category[a]	0.004 (-0.009,0.018)	0.012 (-0.008,0.033)	-0.009 (-0.021,0.004)	-0.012 (-0.035,0.011)
Change low to high category[a]	-0.012 (-0.027,0.003)	-0.003 (-0.026,0.020)	-0.014 (-0.027,-0.0003)*	0.003 (-0.022,0.027)
Change low to very high category[a]	-0.014 (-0.031,0.002)	0.028 (-0.0005,0.057)	-0.018 (-0.034,-0.002)*	-0.006 (-0.033,0.022)
Change normal to hyperuricemia	-0.005 (-0.021,0.011)	0.022 (-0.002,0.046)*	-0.007 (-0.012,0.004)	-0.012 (-0.031,0.007)
		**Lumbar spine**		
**Model 1**				
**Uric acid, mg/dL**	-0.011 (-0.019,-0.003)**	-0.002 (-0.008,0.005)	-0.012 (-0.019,-0.006)***	0.002 (-0.005,0.009)
Change low to medium category[a]	-0.0003 (-0.021,0.020)	0.007 (-0.012,0.025)	-0.020 (-0.036,-0.003)*	0.0004 (-0.022,0.023)
Change low to high category[a]	-0.010 (-0.032,0.013)	-0.016 (-0.036,0.004)	-0.034 (-0.052,-0.016)***	0.004 (-0.020,0.028)
Change low to very high category[a]	-0.024 (-0.048,0.001)	-0.001 (-0.027,0.024)	-0.035 (-0.056,-0.015)**	-0.001 (-0.028,0.026)
Change normal to hyperuricemia	-0.026 (-0.051,-0.002)*	0.005 (-0.017,0.028)	-0.009 (-0.024,0.006)	0.0005 (-0.018,0.019)
**Model 2**				
**Uric acid, mg/dL**	-0.010 (-0.018,-0.002)*	-0.001 (-0.008,0.005)	-0.011 (-0.018,-0.005)***	0.002 (-0.005,0.009)
Change low to medium category[a]	0.00009 (-0.020,0.021)	0.006 (-0.012,0.025)	-0.019 (-0.035,0.002)	0.001 (-0.022,0.024)
Change low to high category[a]	-0.009 (-0.031,0.014)	-0.017 (-0.038,0.004)	-0.033 (-0.050,-0.015)***	0.009 (-0.016,0.033)

Change low to very high category[a]	-0.023 (-0.048,0.002)	0.0007 (-0.026,0.027)	-0.032 (-0.053,-0.011)**	-0.0007 (-0.028,0.027)
Change normal to hyperuricemia	-0.022 (-0.047,0.002)	0.007 (-0.016,0.029)	-0.006 (-0.021,0.009)	-0.0004 (-0.019,0.018)
**Model 3**				
**Uric acid, mg/dL**	-0.010 (-0.018,-0.002)*	0.00001 (-0.007,0.007)	-0.010 (-0.017,-0.004)***	0.0004 (-0.007,0.007)
Change low to medium category[a]	0.0001 (-0.020,0.021)	0.014 (-0.005,0.034)	-0.016 (-0.032,0.0006)	0.0004 (-0.022,0.023)
Change low to high category[a]	-0.009 (-0.031,0.014)	-0.011 (-0.033,0.011)	-0.030 (-0.048,-0.013)**	0.004 (-0.020,0.029)
Change low to very high category[a]	-0.023 (-0.048,0.003)	0.008 (-0.020,0.035)	-0.029 (-0.050,-0.008)**	-0.004 (-0.031,0.023)
Change normal to hyperuricemia	-0.023 (-0.047,0.002)	0.007 (-0.015,0.030)	-0.005 (-0.020,0.009)	-0.004 (-0.023,0.015)

[a] Categories defined by quartiles. Hyperuricemia was defined as 7.0 mg/dl among males and 5.7 mg/dl among females. * p<0.05, ** p<0.01, *** p<0.001. Model 1: unadjusted. Model 2: Adjusted by age, body mass index categories, smoking status, leisure time physical activity, hormone replacement therapy, CKD-EPI equation and diabetes. Model 3: included the additional predictors from model 2 plus adjustment for calcium intake, calcium supplements and dietary inflammatory index.

Supplementary Table S4. Sensitivity analysis: Cross-sectional association between uric acid and BMD at baseline by sex and age groups (<50 and ≥50 years old)

	<50 years		≥50 years	
	**Females (n=628)** β (% 95 CI)	**Males (n=244)** β (% 95 CI)	**Females (n=423)** β (% 95 CI)	**Males (n=128)** β (% 95 CI)
		**Hip BMD**		
**Adjusted Model**				
**Uric acid, mg/dL**	0.008(-0.0006,0.0016)	0.004(-0.009,0.017)	0.012(-0.003,-0.022)**	-0.012(-0.032,0.008)
Low[a]	0.0	0.0	0.0	0.0
Medium category[a]	0.013(-0.011,0.038)	0.015(-0.034,0.065)	0.011(-0.020,0.042)	-0.011(-0.084,0.062)
High category[a]	0.012(-0.015,0.038)	-0.003(-0.055,0.049)	0.019(-0.013,0.050)	-0.0007(-0.075,0.074)
Very high category[a]	0.022(-0.005,0.048)	0.016(-0.037,0.069)	0.037(0.004,0.071)*	-0.030(-0.109,0.050)
Normal	0.0	0.0	0.0	0.0
Hyperuricemia	0.031(-0.0007,0.062)	0.011(-0.033,0.055)	0.038(0.013,0.063)**	-0.024(-0.091,0.044)
		**Femoral neck BMD**		
**Adjusted Model**				
**Uric acid, mg/dL**	0.008(-0.0003,0.016)	0.008(-0.005,0.021)	0.010(0.0002,0.019)*	-0.013(-0.032,0.007)
Low[a]	0.0	0.0	0.0	0.0
Medium category[a]	0.027(0.002,0.052)	-0.003(-0.053,0.049)	0.009(-0.021,0.039)	-0.011(-0.085,0.062)
High category[a]	0.030(0.004,0.056)*	-0.004(-0.058,0.049)	0.007(-0.023,0.038)	-0.002(-0.073,0.077)
Very high category[a]	0.026(-0.0002,0.052)	0.024(-0.030,0.0769	0.031(-0.001,0.063)	-0.037(-0.117,0.043)
Normal	0.0	0.0	0.0	0.0
Hyperuricemia	0.010(-0.021,0.042)	0.024(-0.022,0.069)	0.032(0.008,0.056)**	-0.037(-0.105,0.031)
		**Lumbar spine**		
**Adjusted Model**				
**Uric acid, mg/dL**	-0.002(-0.011,0.008)	0.004(-0.010,0.017)	0.013(0.002,0.026)*	-0.001(-0.027,0.024)

Low[a]	0.0	0.0	0.0	0.0
Medium category[a]	-0.002(-0.032,0.027)	0.010(-0.042,0.063)	0.020(-0.020,0.060)	0.060(-0.033,0.155)
High category[a]	0.006(-0.025,0.037)	0.043(-0.012,0.098)	0.0062(-0.034,0.047)	0.038(-0.057,0.0133)
Very high category[a]	-0.011(-0.042,0.020)	0.013(-0.044,0.069)	0.050(0.007,0.093)*	0.018(-0.084,0.120)
Normal	0.0	0.0	0.0	0.0
Hyperuricemia	-0.009(-0.047,0.028)	-0.010(-0.057,0.038)	0.035(0.0028,0.068)*	-0.022(-0.109,0.065)

[a] Categories defined by quartiles. Hyperuricemia was defined as 7.0 mg/dl among males and 5.7 mg/dl among females. * $p<0.05$, ** $p<0.01$. Adjusted by age, body mass index categories, smoking status, leisure time physical activity, hormone replacement therapy, CKD-EPI equation, diabetes, calcium intake, calcium supplements and dietary inflammatory index.

Supplementary Table S5. Sensitivity analysis: BMD change according to Changes in uric acid between baseline and follow-up by sex and age groups (<50 and ≥50 years old)

	<50 years		≥50 years	
	**Females (n=628)**	**Males (n=244)**	**Females (n=423)**	**Males (n=128)**
	**β (% 95 CI)**	**β (% 95 CI)**	**β (% 95 CI)**	**β (% 95 CI)**
**Hip BMD**				
**Adjusted Model**				
**Uric acid, mg/dL**	-0.008(-0.012,-0.004)***	0.002(-0.002,0.007)	-0.002(-0.007,0.003)	0.003(-0.004,0.010)
Change low to medium category[a]	0.003(-0.007,0.014)	0.012(-0.0008,0.026)	-0.005(-0.018,0.009)	-0.003(-0.027,0.021)
Change low to high category[a]	-0.017(-0.028,-0.005)**	0.007(-0.007,0.022)	-0.008(-0.022,0.007)	0.012(-0.013,0.036)
Change low to very high category[a]	-0.016(-0.029,-0.004)**	0.023(0.005,0.041)**	-0.0005(-0.018,0.017)	0.005(-0.022,0.032)
Change normal to hyperuricemia	-0.009(-0.020,0.002)	0.009(-0.006,0.024)	0.007(-0.011,0.012)	-0.004(-0.023,0.015)
**Femoral neck BMD**				
**Adjusted Model**				
**Uric acid, mg/dL**	-0.009(-0.014,-0.005)***	0.0007(-0.005,0.007)	-0.003(-0.009,0.002)	-0.001(-0.010,0.007)
Change low to medium category[a]	0.002(-0.010,0.014)	0.013(-0.004,0.030)	-0.002(-0.017,0.013)	-0.002(-0.032,0.028)
Change low to high category[a]	-0.017(-0.031,-0.004)*	0.003(-0.016,0.022)	-0.012(-0.028,0.005)	0.011(-0.019,0.042)
Change low to very high category[a]	-0.022(-0.036,-0.008)**	0.029(0.006,0.052)*	-0.0007(0.020,0.018)	-0.012(-0.045,0.022)
Change normal to hyperuricemia	-0.017(-0.030,-0.004)**	0.013(-0.006,0.032)	0.003(-0.010,0.016)	-0.019(-0.041,0.004)
**Lumbar spine**				
**Adjusted Model**				
**Uric acid, mg/dL**	-0.019(-0.026,-0.011)***	-0.0001(-0.006,0.006)	0.0003(-0.004,0.010)	0.0001(-0.009,0.091)
Change low to medium category[a]	0.0005(-0.021,0.018)	0.007(-0.009,0.023)	0.010(-0.027,0.008)	0.004(-0.027, 0.036)
Change low to high category[a]	-0.024(-0.044,-0.003)*	-0.006(-0.024,0.012)	-0.016(-0.035,0.004)	0.0002(-0.032, 0.032)

Change low to very high category[a]	-0.040(-0.062,-0.018)***	0.002(-0.020,0.023)	0.008(-0.015,0.030)	-0.003(-0.038,0.033)
Change normal to hyperuricemia	-0.040(-0.061,-0.019)***	0.003(-0.014,0.021)	0.016(0.0005,0.031)	-0.006(-0.030,0.018)

[a] Categories defined by quartiles. Hyperuricemia was defined as 7.0 mg/dl among males and 5.7 mg/dl among females. * $p<0.05$, ** $p<0.01$, *** $p<0.001$. Adjusted by age, body mass index categories, smoking status, leisure time physical activity, hormone replacement therapy, CKD-EPI equation, diabetes, calcium intake, calcium supplements and dietary inflammatory index.
